# Microwave-Assisted Synthesis of New Substituted Anilides of Quinaldic Acid ^†^

**DOI:** 10.3390/molecules17021292

**Published:** 2012-01-31

**Authors:** Pavel Bobal, Josef Sujan, Jan Otevrel, Ales Imramovsky, Zdenka Padelkova, Josef Jampilek

**Affiliations:** 1 Department of Chemical Drugs, Faculty of Pharmacy, University of Veterinary and Pharmaceutical Sciences, Palackeho 1/3, 612 42 Brno, Czech Republic; 2 Institute of Organic Chemistry and Technology, Faculty of Chemical Technology, University of Pardubice, Studentska 573, 532 10 Pardubice, Czech Republic; 3 Department of General and Inorganic Chemistry, Faculty of Chemical Technology, University of Pardubice, Studentska 573, 532 10 Pardubice, Czech Republic

**Keywords:** microwave-assisted synthesis, amide formation, quinoline-2-carboxanilides, X-ray diffraction, monoclinic lattice

## Abstract

In this study a one step method for the preparation of substituted anilides of quinoline-2-carboxylic acid was developed. This efficient innovative approach is based on the direct reaction of an acid or ester with substituted anilines using microwave irradiation. The optimized method was used for the synthesis of a series of eighteen substituted quinoline-2-carboxanilides. The molecular structure of *N*-(4-bromophenyl)quinoline-2-carboxamide as a model compound was determined by single-crystal X-ray diffraction. It crystallizes in the monoclinic space group with four molecules within the unit cell and the total structure of the compound can be described as “a slightly screwed boat”.

## 1. Introduction

Derivatives of quinoline and its isosteres and analogues are remarkable compounds with many different kinds of biological effects. A number of quinoline-related compounds express antifungal [1,2,3,4], antibacterial [5,6,7], antiviral [8,9,10], antineoplastic [11,12,13,14,15] and other activities [16,17,18].

The stable and polar amide group is an important functionality among the organic substances present in common natural materials like proteins. Moreover, this moiety is found in many synthetic compounds, such as active pharmaceutical ingredients (APIs) or prodrugs [19]. Due to these facts, the amide group is a subject of high interest in drug design and discovery, therefore formation of amides from amines and carboxylic acids and their derivatives is one of the most described transformations. The formation of the amide bond requires activation of a carboxylic acid functional group. The most common methods involve either its activation through acyl chlorides, anhydrides, active esters and other reactive carboxylic acid derivatives or *in situ* activation by using a large family of various coupling reagents. Although both approaches usually afford satisfactory results, they often need expensive coupling reagents or lead to the formation of by-products requiring further separations [20,21].

Microwave-assisted organic synthesis has been successfully applied in organic and medicinal chemistry over the last decades. The use of microwave irradiation as non-conventional energy source to simplify and improve classic organic reactions has become a very popular method, because it often leads to higher yields, improved conversions, clean product formation and shorter reaction times [22,23,24,25,26,27,28].

## 2. Results and Discussion

### 2.1. Chemistry

**Scheme 1:**
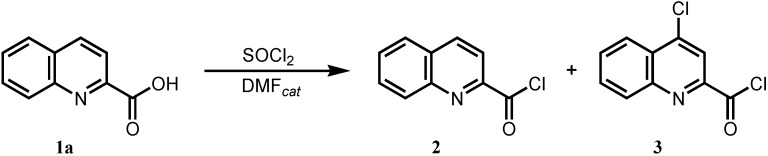
Standard synthesis of quinoline-2-carbonyl chloride (**2**) together with 4-chloroquinoline-2-carbonyl chloride (**3**).

Substituted quinoline-2-carboxanilides were initially synthesized from quinoline-2-carboxylic acid and corresponding substituted anilines. The activation of carboxylic function was carried out by using an excess of thionyl chloride and catalytic amount of dimethylformamide in standard manner. When this type of activation was performed, in addition to desired acyl chloride **2** undesired chlorinated by-product **3** formed by chlorine attack on position 4 of the quinoline ring was observed (Scheme 1).

It is well-known that pyridine ring chlorination occurs when pyridinecarboxylic acid is treated with thionyl chloride in the presence of a small amount of anhydrous dimethylformamide. The formed so-called Vilsmeier reagent is well described and used for formation of 4-chloropyridines as intermediates in the synthesis of the multiple kinase inhibitor sorafenib [29]. A method for direct chlorination of quinoline in position 4 has not been described in the literature so far. On the other hand, formation of 4-chloroquinoline derivative hampered our efforts to prepare pure quinoline-2-carboxanilides. When anilides were prepared from the mixture of acyl chlorides **2** and **3** (20–30%), there was no option to separate them other than chromatography. Attempts to optimize reaction conditions (temperature, ratio of reagents, reaction time, *etc*.) to reduce the amount of undesired intermediate **3** all failed.

**Scheme 2:**
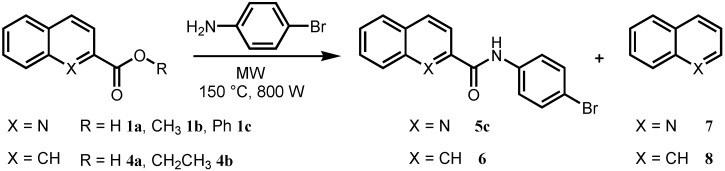
Optimization process of synthesis of *N*-(4-bromophenyl)quinoline-2-carboxamide (**5c**) and *N*-(4-bromophenyl)naphthalene-2-carboxamide (**6**) under microwave irradiation.

Another possibility for the synthesis of the desired quinoline-2-carboxanilides is to perform a direct amidation process starting from an acid or ester precursor and substituted anilines under microwave irradiation [28,30]. Therefore the reaction was performed in a StartSynth microwave reactor using different starting materials. The method was tested on following compounds: quinoline-2-carboxylic acid (**1a**), 2-naphthoic acid (**4a**) and their esters methyl quinoline-2-carboxylate (**1b**), phenyl quinoline-2-carboxylate (**1c**) and ethyl 2-naphthoate (**4b**). All those compounds reacted with 4-bromaniline as a model amine to yield the desired products *N*-(4-bromophenyl)quinoline-2-carboxamide (**5c**) and *N*-(4-bromophenyl)naphthalene-2-carboxamide (**6**), see Scheme 2. The synthesis was performed under various conditions: solvent-free, in dimethylformamide (DMF) or in chlorobenzene (PhCl). All the reactions were performed without or with homo/heterogeneous catalysts: *p*-toluenesulfonic acid (PTSA), potassium *tert*-butoxide (*t*BuOK), silica gel and KF/Al_2_O_3_. Microwave output power of the reactor in all experiments was set to 800 W. The reaction temperature was 150 °C in order to shift the equilibrium by water removal. The ratio of an acid derivative and amine was 1:1.5. The reactions were irradiated up to maximum 2 h and were monitored by HPLC analysis. The results are summarized in Table 1.

**Table 1:** Reaction of 4-bromoaniline with quinoline-2-carboxylic (**1a**) and naphthalene-2-carboxylic (**4a**) acids and their derivatives **1b**, **1c**, **4b** under microwave irradiation.

**Comp.**	**Solvent**	**Catalyst**	**Conversion after 0.5 h**		**Conversion after 1 h**		**Conversion after 2 h**	
			Amide **5c** or **6**	Product **7** or **8**	Amide **5c** or **6**	Product **7** or **8**	Amide **5c** or **6**	Product **7** or **8**
**1a**	–	–	57%	37%	–*^a^*	– *^a^*	–*^a^*	–*^a^*
**4a**	–	–	–	–	–	–	9%	–
**1b**	–	–	6%	–	21%	–	51%	–
**4b**	–	–	–	–	–	–	–	–
**1c**	–	–	96%	–	100%	–	–*^e^*	–*^e^*
**1a**	DMF	–	–	–	–	–	–	–
**4a**	DMF	–	–	–	–	–	–	–
**1b**	DMF	–	–	–	–	–	–	–
**4b**	DMF	–	–	–	–	–	–	–
**1a**	PhCl	–	–	–	–	–	–	–
**4a**	PhCl	–	–	–	–	–	7%	–
**1b**	PhCl	–	–	–	–	–	–	–
**4b**	PhCl	–	–	–	–	–	–	–
**1a**	–	PTSA	53%	46%	55%	44%	33% *^b^*	67% *^b^*
**4a**	–	PTSA	20%	–	46%	–	100% *^a^*	– *^a^*
**1b**	–	PTSA	35%	–	– *^a^*	– *^a^*	–*^a^*	–*^a^*
**4b**	–	PTSA	–	–	–	–	–	–
**1a**	DMF	PTSA	–*^c^*	100%	–*^c^*	100%	–*^c^*	100%
**1b**	DMF	PTSA	–	–	–	–	–*^c^*	–
**4b**	DMF	PTSA	–	–	–	–	–	–
**1a**	PhCl	PTSA	19%	24%	28%	25%	32%	30%
**4a**	PhCl	PTSA	–	–	–	–	–	–
**1b**	PhCl	PTSA	20%	–	25%	–	34%	–
**4b**	PhCl	PTSA	–	–	–	–	–	–
**1a**	–	*t*BuOK	59%	41%	61%	39%	62%	38%
**4a**	–	*t*BuOK	11%	–	20%	–	44%	–
**1b**	–	*t*BuOK	26%	74%	43% *^a^*	56% *^a^*	–*^a^*	–*^a^*
**4b**	–	*t*BuOK	–	–	–	–	–	–
**1a**	DMF	*t*BuOK	–	100%	–	100%	–	100%
**4a**	DMF	*t*BuOK	–	–	–	–	–	–
**1b**	DMF	*t*BuOK	– *^c^*	–	–*^c^*	–	–*^c^*	–
**4b**	DMF	*t*BuOK	–	–	–	–	–	–
**1a**	PhCl	*t*BuOK	13%	27%	30%	34%	35%	41%
**4a**	PhCl	*t*BuOK	–	–	–	–	–	–
**1b**	PhCl	*t*BuOK	17%	–	18%	–	16% *^b^*	–
**4b**	PhCl	*t*BuOK	–	–	–	–	–	–
**1a**	–	silica gel	63%	37%	17%	83%	53%	47%
**4a**	–	silica gel	–	–	22%	–	100% *^d^*	–
**1b**	–	silica gel	11%	–	30%	–	19%	19%
**4b**	–	silica gel	–	–	–	–	–	–
**1a**	DMF	silica gel	– *^c^*	–	– *^c^*	–	–*^c^*	–
**4a**	DMF	silica gel	–	–	–	–	–	–
**1b**	DMF	silica gel	–	–	–	–	–*^c^*	–
**4b**	DMF	silica gel	–	–	–	–	–	–
**1a**	PhCl	silica gel	–*^c^*	26%	9%	23%	68%	8%
**4a**	PhCl	silica gel	–	–	–	–	–	–
**1b**	PhCl	silica gel	–	–	6%	–	9%	–
**4b**	PhCl	silica gel	–	–	–	–	–	–
**1a**	–	KF/Al_2_O_3_	–	–	–	–	90%	8%
**4a**	–	KF/Al_2_O_3_	–	–	–*^c^*	–	–*^c^*	–
**1b**	–	KF/Al_2_O_3_	10%	–	18%	–	35%	–
**4b**	–	KF/Al_2_O_3_	–	–	–	–	–	–
**1a**	DMF	KF/Al_2_O_3_	–	73%	–	89%	–	98%
**4a**	DMF	KF/Al_2_O_3_	–	–	–	–	–	–
**1b**	DMF	KF/Al_2_O_3_	–	–	–	–	–	–
**4b**	DMF	KF/Al_2_O_3_	–	–	–	–	–	–
**1a**	PhCl	KF/Al_2_O_3_	–	–	7%	24%	20%	20%
**4a**	PhCl	KF/Al_2_O_3_	–	–	–	–	–	–
**1b**	PhCl	KF/Al_2_O_3_	–	–	–	–	–*^c^*	–
**4b**	PhCl	KF/Al_2_O_3_	–	–	–	–	–	–

*^a^* decomposition; *^b^* partial decomposition; *^c^* traces of product; *^d^* many impurities; *^e^* not performed.

From the first tests it was already evident that the direct amidation of 2-quinaldic acid (**1a**) is hampered by formation of decarboxylated product—quinoline (**7**). It was determined that when the reaction was carried out in DMF and catalysed either with PTSA or *t*BuOK, only decarboxylated compound **7** was produced. On the other hand, in case of 2-naphthoic acid (**4a**) there no traces of decarboxylated product **8** (naphthalene) were observed.

**Scheme 3:**
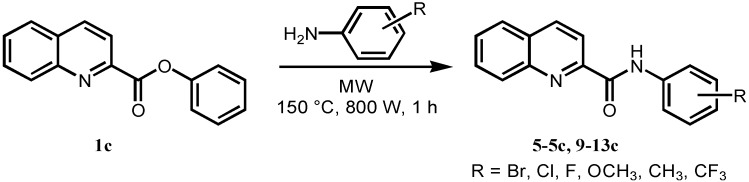
Optimized microwave-assisted synthesis of substituted quinoline-2-carboxanilides **5–5c**, **9–13c**.

When the reaction was performed in solvents like DMF or chlorobenzene, it generally did not lead to any improvement. Although the use of methyl ester **1b** suppressed decarboxylation, it did not enhance reactivity towards the amides significantly. The same applies to ethyl ester **4b**. The results showed that, in almost all cases, the reactions did not proceed cleanly, and the formation of side products and impurities was noticed. Finally, utilization of phenyl ester **1c** in reaction with 4-bromo-aniline under microwave irradiation in solvent-free conditions showed spectacular acceleration, high conversion in relatively short reaction time and high product purity. Having optimized the substrate structure and the conditions in hand, the scope of the procedure was consequently evaluated by varying the aniline. Eighteen commercially available ring-substituted anilines were explored as reaction partners to phenyl ester **1c** and very good yields (61–89%) and satisfactory purities of products **5**–**5c** and **9**–**13c** were obtained. All of the studied compounds were prepared according to Scheme 3.

### 2.2. Crystallography

**Figure 1:**
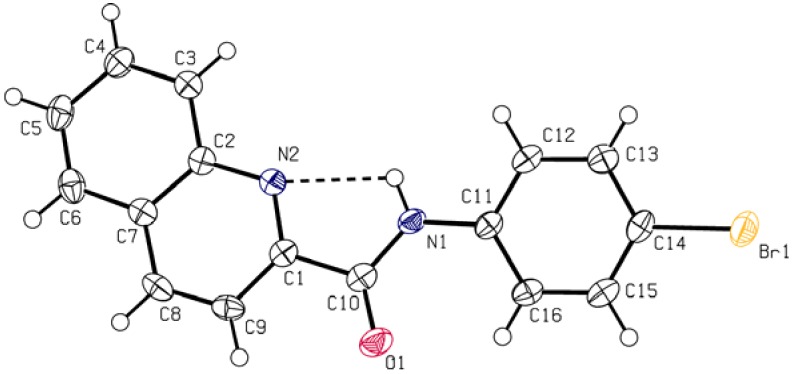
Molecular structure (ORTEP 50% probability level) with *H*-bonding interaction (N(1)-H(1)···N(2) 2.663(3) Å) found in solid state structure. Selected interatomic distances (Ǻ) and angles (°): Br1 C14 1.897(3), C11 N1 1.404(4), N1 C10 1.359(4), C10 C1 1.509(4), C1 N2 1.320(4), N2 C2 1.370(4), O1 C10 1.218(3); C11 N1 C10 127.9(2), N1 C10 O1 125.4(3), N1 C10 C1 113.4(2).

*N*-(4-Bromophenyl)quinoline-2-carboxamide (**5c**; Figure 1), crystallizes in the monoclinic space group *P*2_1_/c with four molecules within the unit cell. 

**Figure 2:**
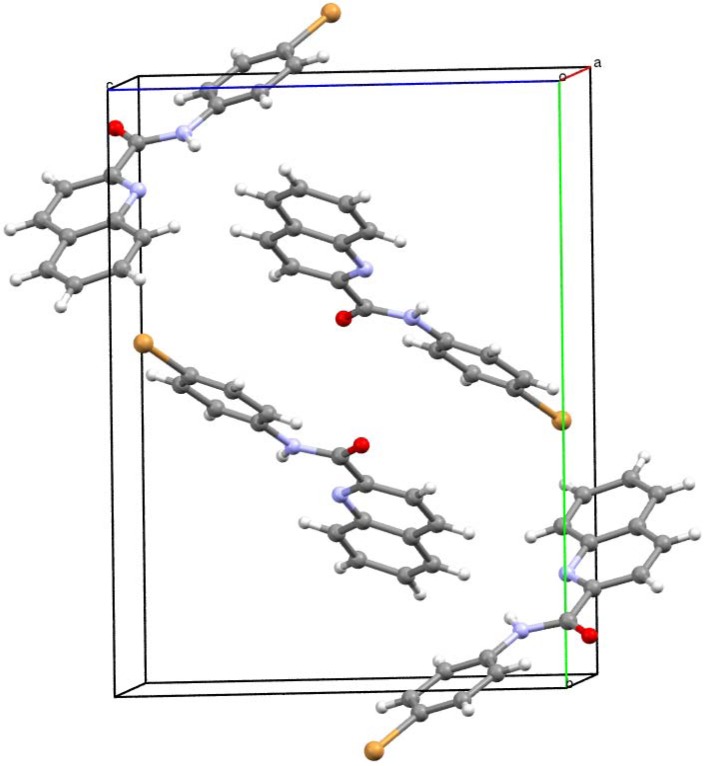
Supramolecular architecture in X, view along the *a* axis.

The total structure of **5c** can be described as a slightly screwed boat with no intermolecular hydrogen bonding. The intramolecular N1-H1···N2 contact is present along with another short contacts forming the 3D structure (Figure 2), instead of a stairs-like supramolecular architecture typical for previously reported members of the families of *N*-(4-halophenyl)quinoline-2-carboxamides [31] or *N*-(4-halophenyl)pyridine-2-carboxamides [32,33,34,35].

These molecules have typically 2D-layered structures without *H*-bridges and even π-π stacking. On the other hand, the structure of **5c** reveals a weak interaction between the C=O moiety and the coplanar aromatic system of the proximate molecule. The contact between the bromine atom and the perpendicularly oriented π-system of the quinoline ring is probably responsible for the 3D arrangement of molecules.

## 3. Experimental Section

### 3.1. General

All chemicals were reagent grade and were purchased from Sigma-Aldrich and Acros. TLC analysis was performed on precoated 60 F_254_ plates (Merck, Darmstadt, Germany). Compounds were visualized by UV light (254 nm) and evaluated in iodine vapour. Small-scale microwave-assisted synthesis was carried out in a StartSynth multimode microwave instrument producing controlled irradiation at 2.45 GHz (Milestone S.r.l., Sorisole, Italy). The instrument is equipped with an industrial magnetron and a microwave diffuser located above the microwave chamber, with continuous microwave output power from 0 to 1400 W. Reaction times refer to hold times at the temperatures indicated, not to total irradiation times. The temperature was measured with an IR sensor on the outside of the reaction vessel. HPLC monitoring analyses were performed on an Agilent 1200 series HPLC system equipped with a diode array detection (DAD) system, a quarternary model pump and an automatic injector (Agilent Technologies, Santa Clara, CA, USA). ChemStation Chromatography Software was used for data acquisition. Acetonitrile (HPLC grade, Sigma-Aldrich, 50.0%) and H_2_O (HPLC grade, Sigma-Aldrich, 50.0%) were used as the mobile phase. A Gemini-NX 100 (Phenomenex, Torrance, CA, USA), C18 3 μm, 2.0 × 100 mm chromatographic column was used with a total flow of 0.2 mL/min, an injection volume of 5 μL and a column temperature of 35 °C. A detection wavelength of 230 nm was chosen. The melting points were determined on a Boetius PHMK 05 (VEB Kombinat Nagema, Radebeul, Germany) and are uncorrected. Infrared (IR) spectra were recorded on a Smart MIRacle™ ATR ZnSe for Nicolet™ Impact 410 FT-IR Spectrometer (Thermo Scientific, Waltham, MA, USA). The spectra were obtained by accumulation of 256 scans with 2 cm^−1^ resolution in the region of 4,000–600 cm^−1^. All ^1^H and ^13^C-NMR spectra were recorded in DMSO-*d_6_* solutions at ambient temperature on a Bruker Avance III 400 MHz spectrometer (Karlsruhe, Bruker, Germany, 400 MHz for ^1^H, 100 MHz for ^13^C). Chemical shifts are reported in ppm (δ). Proton chemical shifts in DMSO-*d*_6_ are related to the middle of the solvent multiplet (δ = 2.50). ^13^C-NMR spectra were measured using APT pulse sequences. Carbon chemical shifts are referenced to the middle of the solvent multiplet (δ = 39.5 in DMSO-*d*_6_). Mass spectra were measured using a LTQ Orbitrap Hybrid Mass Spectrometer (Thermo Electron Corporation, Waltham, MA, USA) with direct injection into an APCI source (400 °C) in the positive mode.

### 3.2. Synthesis

#### 3.2.1. Procedure for Classical Synthesis of Ring-substituted Quinoline- and 4-chloroquinoline-2-carboxanilides

Quinoline-2-carboxylic acid (**1a**, 1.0 g, 5.8 mmol) was suspended in thionyl chloride (2.1 mL, 3.4 g, 28.9 mmol) at room temperature, and DMF (2 drops) was added. The mixture was refluxed for about 3 h and then evaporated to dryness. The residue was used directly in the next step. Into the solution of 2-quinaldic acid chloride in dry toluene (15 mL), triethylamine (4.5 mL, 2.92 g, 32.5 mmol) and the corresponding substituted aniline (5.8 mmol) were added dropwise. The mixture was stirred at room temperature for 24 h, after which the solvent was removed under reduced pressure. The residue was extracted with CHCl_3_. The combined organic layers were washed with water and saturated aqueous solution of NaHCO_3_ and dried over anhydrous MgSO_4_. The solvent was evaporated to dryness under reduced pressure. The crude product and its 4-chloro derivative were isolated by flash chromatography (*n*-hexane/EtOAc 3: 2) and recrystallized from isopropanol or EtOAc.

#### 3.2.2. Procedure for the Optimization of Microwave-assisted Synthesis

Quinoline-2-carboxylic acid (**1a**) or naphthalene-2-carboxylic acid (**4a**) or their esters **1b**, **1c**, **4b** (1.7 mmol) and 4-bromoaniline (0.45 g, 2.6 mmol) were mixed in 10 mL round bottom flask and placed to the microwave reactor. Outlet of the reaction flask was connected with a tube attached to a condenser outside of the microwave reactor. The microwave output power was selected to maximum 800 W. The stirred reaction mixture was preheated to 150 °C by microwave irradiation and let to react at the same temperature for 2 h. The reaction was monitored by HPLC in time periods: 0.5 h, 1 h and 2 h. The results are presented in Table 1.

#### 3.2.3. General Procedure for Microwave-assisted Synthesis of Ring-substituted Quinoline-2-carboxanilides

Phenyl quinoline-2-carboxylate (**1c**, 250 mg, 1.0 mmol) and substituted aniline (1.5 mmol) were mixed in 10 mL round bottom flask and placed to the microwave reactor. Outlet of the reaction flask was connected with a tube attached to a condenser outside of the microwave reactor. The microwave output power was selected to maximum 800 W in order to avoid the observed pyrolysis problems. The stirred reaction mixture was preheated to 150 °C by microwave irradiation and let to react at the same temperature for 1 h. After cooling, the reaction mixture was diluted with chloroform (20 mL), washed with saturated sodium bicarbonate solution (2 × 10 mL) and brine (10 mL). The organic phase was then dried over anhydrous Na_2_SO_4_ and the solvent was removed under reduced pressure. The crude product was recrystallized from isopropanol to yield pure substituted quinoline-2-carboxanilides **5**–**5c**, **9**–**13c**.

*N-(2-Bromophenyl)quinoline-2-carboxamide* (**5a**). Yield 61%; Mp. 134–135 °C; IR (Zn/Se ATR, cm^−1^): 3277*w*, 1689*s*, 1588*m*, 1579*m*, 1543*m*, 1530*s*, 1496*m*, 1440*m*, 1427*m*, 1302*w*, 1132*w*, 1204*m*, 908*w*, 842*m*, 768*s*, 736*m*, 698*m*; ^1^H-NMR (DMSO-*d*_6_), δ: 10.82 (bs, 1H), 8.60 (d, *J* = 8.5 Hz, 1H), 8.44 (d, *J* = 8.3 Hz, 1H), 8.23 (d, *J* = 8.5 Hz, 1H), 8.13 (d, *J* = 8.5 Hz, 1H), 8.07 (d, *J* = 8.3 Hz, 1H), 7.87 (t, *J* = 7.5 Hz, 1H), 7.64–7.77 (m, 2H), 7.44 (t, *J* = 7.8 Hz, 1H), 7.10 (t, *J* = 7.7 Hz, 1H); ^13^C-NMR (DMSO-*d*_6_), δ: 161.61, 148.71, 145.50, 138.60, 135.46, 132.58, 130.83, 129.26, 129.13, 128.55, 128.53, 128.08, 125.74, 121.39, 118.19, 114.08; HR-MS: for C_16_H_12_BrN_2_O [M+H]^+^ calculated 327.0133 *m/z*, found 327.0138 *m/z*.

*N-(3-Bromophenyl)quinoline-2-carboxamide* (**5b**). Yield 75%; Mp. 139–140 °C; IR (Zn/Se ATR, cm^−1^): 3318*w*, 1687*m*, 1581*m*, 1519*m*, 1478*w*, 1408*m*, 1296*w*, 1124*m*, 1067*w*, 912*w*, 847*m*, 764*s*, 685*m*; ^1^H-NMR (DMSO-*d*_6_), δ: 10.89 (bs, 1H), 8.60 (d, *J* = 8.3 Hz, 1H), 8.19–8.32 (m, 3H), 8.09 (d, *J* = 8.0 Hz, 1H), 7.96 (d, *J* = 7.5 Hz, 1H), 7.87–7.93 (m, 1H), 7.68–7.78 (m, 1H), 7.27–7.40 (m, 2H); ^13^C-NMR (DMSO-*d*_6_), δ: 163.02, 149.67, 145.86, 139.98, 138.21, 130.69, 130.67, 129.32, 128.97, 128.44, 128.14, 126.59, 122.66, 121.55, 119.14, 118.77; HR-MS: for C_16_H_12_BrN_2_O [M+H]^+^ calculated 327.0133 *m/z*, found 327.0143 *m/z*.

*N-(4-Bromophenyl)quinoline-2-carboxamide* (**5c**). Yield 88%; Mp. 157–158 °C; IR (Zn/Se ATR, cm^−1^): 3355*w*, 1693*s*, 1581*m*, 1522*s*, 1496*s*, 1423*w*, 1389*m*, 1305*w*, 1120*m*, 1095*w*, 1068*m*, 998*w*, 907*w*, 839*s*, 807*s*, 769*s*, 693*w*; ^1^H-NMR (DMSO-*d*_6_), δ: 10.84 (bs, 1H), 8.58 (d, *J* = 8.5 Hz, 1H), 8.18–8.30 (m, 2H), 8.07 (d, *J* = 8.3 Hz, 1H), 7.95 (d, *J* = 8.8 Hz, 2H), 7.86–7.92 (m, 1H), 7.67–7.78 (m, 1H), 7.56 (d, *J* = 8.8 Hz, 2H); ^13^C-NMR (DMSO-*d*_6_), δ: 162.86, 149.82, 145.87, 138.16, 137.74, 131.53, 130.64, 129.33, 128.94, 128.37, 128.12, 122.28, 118.77, 115.80; HR-MS: for C_16_H_12_BrN_2_O [M+H]^+^ calculated 327.0133 *m/z*, found 327.0129 *m/z*.

*N-(2-Chlorophenyl)quinoline-2-carboxamide* (**9a**) [36]. Yield 70%; Mp. 130–131 °C; ^1^H-NMR (DMSO-*d*_6_), δ: 10.77 (bs, 1H), 8.58 (d, *J* = 8.5 Hz, 1H), 8.43 (d, *J* = 8.0 Hz, 1H), 8.21 (d, *J* = 8.5 Hz, 1H), 8.10 (d, *J* = 8.5 Hz, 1H), 8.05 (d, *J* = 8.3 Hz, 1H), 7.85 (t, *J* = 7.5 Hz, 1H), 7.64–7.75 (m, 1H), 7.54 (d, *J* = 7.8 Hz, 1H), 7.39 (t, *J* = 7.7 Hz, 1H), 7.10–7.24 (m, 1H); ^13^C-NMR (DMSO-*d*_6_), δ: 161.54, 148.70, 145.47, 138.50, 134.21, 130.75, 129.29, 129.20, 129.07, 128.46, 128.00, 127.88, 125.23, 123.38, 121.27, 118.15; HR-MS: for C_16_H_12_ClN_2_O [M+H]^+^ calculated 283.0638 *m/z*, found 283.0652 *m/z*.

*N-(3-Chlorophenyl)quinoline-2-carboxamide* (**9b**) [36]. Yield 80%; Mp. 127–128 °C; ^1^H-NMR (DMSO-*d*_6_), δ: 10.90 (bs, 1H), 8.58 (d, *J* = 8.5 Hz, 1H), 8.18–8.31 (m, 2H), 8.15 (s, 1H), 8.07 (d, *J* = 8.0 Hz, 1H), 7.82–7.97 (m, 2H), 7.66–7.78 (m, 1H), 7.40 (t, *J* = 8.0 Hz, 1H), 7.11–7.23 (m, 1H); ^13^C-NMR (DMSO-*d*_6_), δ: 163.02, 149.67, 145.87, 139.85, 138.20, 133.12, 130.68, 130.36, 129.34, 128.98, 128.43, 128.14, 123.70, 119.82, 118.77; HR-MS: for C_16_H_12_ClN_2_O [M+H]^+^ calculated 283.0638 *m/z*, found 283.0648 *m/z*.

*N-(4-Chlorophenyl)quinoline-2-carboxamide* (**9c**). Yield 80%; Mp. 134–135 °C (Mp. 135–135.5 °C [37]); ^1^H-NMR (DMSO-*d*_6_), δ: 10.88 (bs, 1H), 8.58 (d, *J* = 8.5 Hz, 1H), 8.17–8.30 (m, 2H), 8.08 (d, *J* = 8.0 Hz, 1H), 8.01 (d, *J* = 8.8 Hz, 2H), 7.84–7.93 (m, 1H), 7.68–7.77 (m, 1H), 7.43 (d, *J* = 8.8 Hz, 2H); ^13^C-NMR (DMSO-*d*_6_), δ: 162.87, 149.85, 145.88, 138.16, 137.34, 130.65, 129.34, 128.95, 128.62, 128.37, 128.14, 127.69, 121.92, 118.78; HR-MS: for C_16_H_12_ClN_2_O [M+H]^+^ calculated 283.0638 *m/z*, found 283.0631 *m/z*.

*N-(2-Fluorophenyl)quinoline-2-carboxamide* (**10a**). Yield 63%; Mp. 116–117 °C; IR (Zn/Se ATR, cm^−1^): 3328*w*, 1691*m*, 1615*m*, 1591*w*, 1530*s*, 1504*m*, 1477*w*, 1454*m*, 1428*m*, 1317*w*, 1247*w*, 1185*w*, 1126*m*, 1088*w*, 910*w*, 837*m*, 772*s*, 746*s*, 683*m*; ^1^H-NMR (DMSO-*d*_6_), δ: 10.48 (bs, 1H), 8.57 (d, *J* = 8.5 Hz, 1H), 8.17–8.25 (m, 2H), 8.13 (d, *J* = 8.5 Hz, 1H), 8.05 (d, *J* = 8.0 Hz, 1H), 7.85 (t, *J* = 7.3 Hz, 1H), 7.65–7.76 (m, 1H), 7.28–7.40 (m, 1H), 7.13–7.27 (m, 2H); ^13^C-NMR (DMSO-*d*_6_), δ: 162.00, 153.58 (d, ^1^*J*_FC_ = 244 Hz), 148.95, 145.67, 138.39, 130.76, 129.24, 129.15 (d, ^2^*J*_FC_ = 19.1 Hz), 128.45, 128.08, 125.70 (d, ^3^*J*_FC_ = 11.0 Hz), 125.53 (d, ^3^*J*_FC_ = 7.3 Hz), 124.63 (d, ^4^*J*_FC_ = 3.7 Hz), 122.91, 118.37, 115.43 (d, ^2^*J*_FC_ = 19.1 Hz); HR-MS: for C_16_H_12_FN_2_O [M+H]^+^ calculated 267.0934 *m/z*, found 267.0950 *m/z*.

*N-(3-Fluorophenyl)quinoline-2-carboxamide* (**10b**). Yield 81%; Mp. 126–127 °C; IR (Zn/Se ATR, cm^−1^): 3343*w*, 1690*s*, 1588*m*, 1531*s*, 1504*m*, 1481*s*, 1409*s*, 1170*m*, 1137*m*, 899*m*, 841*s*, 791*m*, 768*s*, 738*m*, 682*s*; ^1^H-NMR (DMSO-*d*_6_), δ: 10.91 (bs, 1H), 8.58 (d, *J* = 8.5 Hz, 1H), 8.16–8.31 (m, 2H), 8.08 (d, *J* = 8.3 Hz, 1H), 7.95 (d, *J* = 11.8 Hz, 1H), 7.86–7.92 (m, 1H), 7.79 (d, *J* = 8.3 Hz, 1H), 7.68–7.75 (m, 1H), 7.35–7.49 (m, 1H), 6.96 (td, *J* = 8.4 Hz, *J* = 2.0 Hz, 1H); ^13^C-NMR (DMSO-*d*_6_), δ: 163.01, 162.15 (d, ^1^*J*_FC_ =241 Hz), 149.73, 145.86, 140.11 (d, ^3^*J*_FC_ =11.0 Hz), 138.18, 130.65, 130.31 (d, ^3^*J*_FC_ = 9.5 Hz), 129.33, 128.97, 128.39, 128.12, 118.77, 116.15 (d, ^4^*J*_FC_ = 2.9 Hz), 110.47 (d, ^2^*J*_FC_ = 21.3 Hz), 107.09 (d, ^2^*J*_FC_ = 26.4 Hz); HR-MS: for C_16_H_12_FN_2_O [M+H]^+^ calculated 267.0934 *m/z*, found 267.0953 *m/z*.

*N-(4-Fluorophenyl)quinoline-2-carboxamide* (**10c**) [36,38]. Yield 81%; Mp. 115–116 °C; ^1^H-NMR (DMSO-*d*_6_), δ: 10.83 (bs, 1H), 8.57 (d, *J* = 8.3 Hz, 1H), 8.17–8.29 (m, 2H), 8.06 (d, *J* = 8.0 Hz, 1H), 7.94–8.02 (m, 2H), 7.87 (td, *J* = 7.7 Hz, *J* = 1.3 Hz, 1H), 7.66–7.76 (m, 1H), 7.17–7.28 (m, 2H); ^13^C-NMR (DMSO-*d*_6_), δ: 162.76, 158.58 (d, ^1^*J*_FC_ = 237 Hz), 150.03, 145.95, 138.18, 134.81 (d, ^4^*J*_FC_ = 2.2 Hz), 130.67, 129.39, 128.98, 128.37, 128.17, 122.31 (d, ^3^*J*_FC_ = 7.3 Hz), 118.83, 115.26 (d, ^2^*J*_FC_ = 22.7 Hz); HR-MS: for C_16_H_12_FN_2_O [M+H]^+^ calculated 267.0934 *m/z*, found 267.0954 *m/z*.

*N-(2-Methoxyphenyl)quinoline-2-carboxamide* (**11a**) [39]. Yield 79%; Mp. 111–112 °C; IR (Zn/Se ATR, cm^−1^): 3382*w*, 1676*s*, 1596*m*, 1532*s*, 1485*w*, 1454*m*, 1426*m*, 1334*w*, 1288*w*, 1253*m*, 1138*m*, 1129*m*, 1093*w*, 1020*s*, 951*w*, 908*m*, 873*w*, 840*m*, 820*w*, 770*s*, 732*s*; ^1^H-NMR (DMSO-*d*_6_), δ: 10.68 (bs, 1H), 8.59 (d, *J* = 8.5 Hz, 1H), 8.49 (d, *J* = 7.8 Hz, 1H), 8.25 (d, *J* = 8.5 Hz, 1H), 8.15 (d, *J* = 8.5 Hz, 1H), 8.07 (d, *J* = 8.3 Hz, 1H), 7.87 (t, *J* = 7.3 Hz, 1H), 7.67–7.75 (m, 1H), 7.11 (d, *J* = 4.0 Hz, 2H), 7.01 (dt, *J* = 8.2 Hz, *J* = 4.2 Hz, 1H), 3.98 (s, 3H); ^13^C-NMR (DMSO-*d*_6_), δ: 161.25, 149.34, 148.51, 145.62, 138.55, 130.82, 129.30, 129.06, 128.44, 128.14, 126.87, 124.25, 120.68, 118.84, 118.27, 110.91, 56.05; HR-MS: for C_17_H_15_N_2_O_2_ [M+H]^+^ calculated 279.1134 *m/z*, found 279.1148 *m/z*.

*N-(3-Methoxyphenyl)quinoline-2-carboxamide* (**11b**). Yield 77%; Mp. 117–118 °C; IR (Zn/Se ATR, cm^−1^): 3352*w*, 1687*m*, 1589*m*, 1524*m*, 1503*m*, 1456*m*, 1425*m*, 1334*w*, 1284*m*, 1203*m*, 1157*m*, 1128*m*, 1049*s*, 906*w*, 876*m*, 854*m*, 823*w*, 798*w*, 762*s*, 740*s*, 685*m*; ^1^H-NMR (DMSO-*d*_6_), δ: 10.73 (bs, 1H), 8.58 (d, *J* = 8.5 Hz, 1H), 8.19–8.32 (m, 2H), 8.07 (d, *J* = 8.0 Hz, 1H), 7.82–7.96 (m, 1H), 7.65–7.79 (m, 2H), 7.59 (dd, *J* = 8.0 Hz, *J* = 1.0 Hz, 1H), 7.29 (t, *J* = 8.2 Hz, 1H), 6.72 (dd, *J* = 8.3 Hz, *J* = 2.01 Hz, 1H), 3.78 (s, 3H); ^13^C-NMR (DMSO-*d*_6_), δ: 162.70, 159.61, 149.99, 145.88, 139.53, 138.23, 130.67, 129.61, 129.37, 128.97, 128.37, 128.16, 118.75, 112.47, 109.68, 105.91, 55.09; HR-MS: for C_17_H_15_N_2_O_2_ [M+H]^+^ calculated 279.1134 *m/z*, found 279.1129 *m/z*.

*N-(4-Methoxyphenyl)quinoline-2-carboxamide* (**11c**) [36]. Yield 84%; Mp. 130–131 °C; ^1^H-NMR (DMSO-*d*_6_), δ: 10.65 (bs, 1H), 8.57 (d, *J* = 8.5 Hz, 1H), 8.24 (d, *J* = 8.5 Hz, 2H), 8.07 (d, *J* = 7.8 Hz, 1H), 7.82–7.95 (m, 3H), 7.63–7.78 (m, 1H), 6.97 (d, *J* = 9.0 Hz, 2H), 3.75 (s, 3H); ^13^C-NMR (DMSO-*d*_6_), δ: 162.28, 155.80, 150.25, 145.90, 138.09, 131.47, 130.59, 129.32, 128.87, 128.22, 128.11, 121.85, 118.73, 113.87, 55.17; HR-MS: for C_17_H_15_N_2_O_2_ [M+H]^+^ calculated 279.1134 *m/z*, found 279.1145 *m/z*.

*N-(2-Methylphenyl)quinoline-2-carboxamide* (**12a**). Yield 71%; Mp. 100–101 °C; IR (Zn/Se ATR, cm^−1^): 3334*w*, 1686*s*, 1587*s*, 1528*s*, 1498*M*, 1454*s*, 1427*s*, 1422*m*, 1373*w*, 1305*m*, 1249*w*, 1201*w*, 1132*m*, 1091*w*, 1040*w*, 1013*w*, 981*w*, 954*m*, 932*w*, 907*m*, 872*m*, 842*s*, 793*w*, 765*s*, 750*s*, 731*s*, 681*s*; ^1^H-NMR (DMSO-*d*_6_), δ: 10.45 (bs, 1H), 8.60 (d, *J* = 8.5 Hz, 1H), 8.24 (d, *J* = 8.5 Hz, 1H), 8.17 (d, *J* = 8.3 Hz, 1H), 8.08 (d, *J* = 8.0 Hz, 1H), 7.95 (d, *J* = 7.8 Hz, 1H), 7.83–7.91 (m, 1H), 7.69–7.77 (m, 1H), 7.22–7.31 (m, 2H), 7.08–7.16 (m, 1H), 2.37 (s, 3H); ^13^C-NMR (DMSO-*d*_6_), δ: 161.92, 149.67, 145.74, 138.35, 135.97, 130.72, 130.42, 130.01, 129.38, 129.01, 128.38, 128.11, 126.40, 124.96, 122.65, 118.49, 17.49; HR-MS: for C_17_H_15_N_2_O [M+H]^+^ calculated 263.1184 *m/z*, found 263.1182 *m/z*.

*N-(3-Methylphenyl)quinoline-2-carboxamide* (**12b**). Yield 65%; Mp. 82–83 °C; IR (Zn/Se ATR, cm^−1^): 3355*w*, 1685*m*, 1592*m*, 1527*s*, 1503*s* 1457*w*, 1424*m*, 1300*w*, 1171*w*, 1125*m*, 908*w*, 852*m*, 773*s*, 740*w*, 690*s*; ^1^H-NMR (DMSO-*d*_6_), δ: 10.66 (bs, 1H), 8.61 (d, *J* = 8.5 Hz, 1H), 8.25 (dd, *J* = 7.9 Hz, *J* = 5.40 Hz, 2H), 8.10 (d, *J* = 8.0 Hz, 1H), 7.90 (t, *J* = 7.5 Hz, 1H), 7.67–7.84 (m, 3H), 7.27 (t, *J* = 7.7 Hz, 1H), 6.96 (d, *J* = 7.3 Hz, 1H), 2.32 (s, 3H); ^13^C-NMR (DMSO-*d*_6_), δ: 162.54, 150.03, 145.88, 138.22, 138.20, 138.03, 130.68, 129.35, 128.94, 128.65, 128.35, 128.15, 124.75, 120.72, 118.70, 117.35, 21.23; HR-MS: for C_17_H_15_N_2_O [M+H]^+^ calculated 263.1184 *m/z*, found 263.1191 *m/z*.

*N-(4-Methylphenyl)quinoline-2-carboxamide* (**12c**). Yield 89%; Mp. 107–108 °C (Mp. 109.5–110 °C [37]); ^1^H-NMR (DMSO-*d*_6_), δ: 10.67 (bs, 1H), 8.59 (d, *J* = 8.5 Hz, 1H), 8.18–8.30 (m, 2H), 8.08 (d, *J* = 8.0 Hz, 1H), 7.79–7.94 (m, 3H), 7.72 (t, *J* = 7.4 Hz, 1H), 7.18 (d, *J* = 8.3 Hz, 2H), 2.27 (s, 3H); ^13^C-NMR (DMSO-*d*_6_), δ: 162.51, 150.17, 145.93, 138.21, 135.86, 133.09, 130.69, 129.39, 129.24, 128.94, 128.35, 128.18, 120.27, 118.77, 20.59; HR-MS: for C_17_H_15_N_2_O [M+H]^+^ calculated 263.1184 *m/z*, found 263.1193 *m/z*.

*N-(2-Trifluoromethylphenyl)quinoline-2-carboxamide* (**13a**). Yield 74%; Mp. 120–121 °C; IR (Zn/Se ATR, cm^−1^): 3316*w*, 1698*s*, 1590*s*, 1537*s*, 1498*w*, 1452*m*, 1423*m*, 1320*m*, 1288*m*, 1244*w*, 1202*w*, 1165*m*, 1124*m*, 1094*m*, 1054*m*, 1026*m*, 953*w*, 906*w*, 871*w*, 836*m*, 792*w*, 763*s*, 676*m*; ^1^H-NMR (DMSO-*d*_6_), δ: 10.78 (bs, 1H), 8.61 (d, *J* = 8.3 Hz, 1H), 8.36 (d, *J* = 8.3 Hz, 1H), 8.23 (d, *J* = 8.3 Hz, 1H), 8.07 (t, *J* = 8.3 Hz, 2H), 7.87 (t, *J* = 7.5 Hz, 1H), 7.64–7.81 (m, 3H), 7.38 (t, *J* = 7.7 Hz, 1H); ^13^C-NMR (DMSO-*d*_6_), δ: 162.05, 148.48, 145.53, 138.74, 135.14, 133.57, 131.01, 129.48 (q, ^2^*J*_FC_ = 37 Hz), 129.21, 129.17, 128.68, 128.16, 126.41 (q, ^3^*J*_FC_ = 5.1 Hz), 125.05, 124.10 (q, ^1^*J*_FC_ = 274 Hz), 123.89 (q, ^3^*J*_FC_ = 5.9 Hz), 118.31; HR-MS: for C_17_H_12_F_3_N_2_O [M+H]^+^ calculated 317.0902 *m/z*, found 317.0891 *m/z*.

*N-(3-Trifluoromethylphenyl)quinoline-2-carboxamide* (**13b**). Yield 71%; Mp. 121–122 °C; IR (Zn/Se ATR, cm^−1^): 3339*w*, 1692*s*, 1614*w*, 1536*m*, 1490*m*, 1424*w*, 1330*s*, 1223*w*, 1166*m*, 1109*s*, 1091*s*, 1065*m*, 952*w*, 933*w*, 874*s*, 844*m*, 08*s*, 771*s*, 744*w*, 698*s*; ^1^H-NMR (DMSO-*d*_6_), δ: 11.08 (bs, 1H), 8.59 (d, *J* = 8.5 Hz, 1H), 8.46 (s, 1H), 8.17–8.31 (m, 3H), 8.08 (d, *J* = 8.0 Hz, 1H), 7.89 (t, *J* = 7.4 Hz, 1 H), 7.68–7.78 (m, 1 H), 7.61 (t, *J* = 8.0 Hz, 1 H), 7.46 (d, *J* = 7.5 Hz, 1H); ^13^C-NMR (DMSO-*d*_6_), δ: 163.26, 149.64, 145.90, 139.23, 138.21, 130.69, 129.86, 129.35 (q, ^2^*J*_FC_ = 32 Hz), 129.34, 129.03, 128.45, 128.16, 124.20 (q, ^1^*J*_FC_ = 273 Hz), 123.91, 120.24 (q, ^3^*J*_FC_ = 3.7 Hz), 118.78, 116.61 (q, ^3^*J*_FC_ = 3.7 Hz); HR-MS: for C_17_H_12_F_3_N_2_O [M+H]^+^ calculated 317.0902 *m/z*, found 317.0892 *m/z*.

*N-(4-Trifluoromethylphenyl)quinoline-2-carboxamide* (**13c**) [38,40]. Yield 87%; Mp. 147‑148 °C; ^1^H-NMR (DMSO-*d*_6_), δ: 11.02 (bs, 1H), 8.59 (d, *J* = 8.3 Hz, 1H), 8.26 (d, *J* = 8.5 Hz, 1H), 8.23 (d, *J* = 8.3 Hz, 1H), 8.19 (d, *J* = 8.5 Hz, 2H), 8.08 (d, *J* = 8.0 Hz, 1H), 7.86–7.93 (m, 1H), 7.69–7.77 (m, 3H); ^13^C-NMR (DMSO-*d*_6_), δ: 163.27, 149.63, 145.88, 141.95, 138.23, 130.69, 129.38, 129.03, 128.48, 128.14, 125.96 (q, ^3^*J*_FC_ = 3.7 Hz), 124.39 (q, ^1^*J*_FC_ = 271 Hz), 124.00 (q, ^2^*J*_FC_ = 32 Hz), 120.29, 118.81; HR-MS: for C_17_H_12_F_3_N_2_O [M+H]^+^ calculated 317.0902 *m/z*, found 317.0890 *m/z*.

### 3.3. Crystallography

The X-ray data for the colourless crystal of **5c** were obtained at 150 K using Oxford Cryostream low-temperature device on a Nonius KappaCCD diffractometer with MoK_α_ radiation (λ = 0.71073 Å), a graphite monochromator and the ϕ and χ scan mode. Data reductions were performed with DENZO-SMN [41]. The absorption was corrected by integration methods [42]. Structures were solved by direct methods (Sir92) [43] and refined by full matrix least-square based on *F^2^* (SHELXL97) [44]. Hydrogen atoms were mostly localized on a difference Fourier map, however to ensure uniformity of the treatment of the crystal, all hydrogen atoms were recalculated into idealized positions (riding model) and assigned temperature factors H_iso_(H) = 1.2 U_eq_(pivot atom) or 1.5 U_eq_ for the methyl moiety with C–H = 0.93 Å for hydrogen atoms in aromatic rings moiety and N–H being 0.86 Å. Crystallographic data for **5c**: C_16_H_11_BrN_2_O, M = 327.18, monoclinic, *P21/c*, *a* = 6.3620(2), *b* = 16.9968(7), *c* = 12.6001(10) Å, β = 105.892(5)°, Z = 4, V = 1310.42(13) Å^3^, D_c_ = 1.658 g·cm^−3^, μ = 3.133 mm^−1^, T_min_/T_max_ = 0.559/0.671; −8 ≤ h ≥ 7, −20 ≤ k ≥ 22, −15 ≤ l ≥ 16; 9690 reflections measured (θ_max_ = 27.4°), 2941 independent (R_int_ = 0.0428), 2246 with *I > 2 σ (I)*, 181 parameters, *S* = 1.159, *R1*(obs. data) = 0.0404, *wR2*(all data) = 0.0672; max., min. residual electron density = 0.324, −0.389 eÅ^−3^. 

Crystallographic data for structural analysis have been deposited with the Cambridge Crystallographic Data Centre under CCDC deposition number: 858014. Copies of this information may be obtained free of charge from the Director, CCDC, 12 Union Road, Cambridge CB2 1EY, UK (fax: +44-1223-336033; e-mail: deposit@ccdc.cam.ac.uk or www: http://www.ccdc.cam.ac.uk).

## 4. Conclusions

A novel microwave-assisted one-pot coupling of phenyl ester of 2-quinaldic acid (**1c**) and ring-substituted anilines was successfully developed. This method provided an efficient approach for the synthesis of substituted quinoline-2-carboxanilides in solvent-free conditions. Interestingly, the reactions were applied and verified to eighteen substituted anilines. Desired carboxanilides were isolated in high yields and purities. In the solid state of *N*-(4-bromophenyl)quinoline-2-carboxamide (**5c**) only typical intramolecular N1-H1···N2 contact was observed. The structure of **5c** shows that there is no strong intermolecular interaction. Compound **5c** is the first structure of this type of compounds with 3D organisation due to several short contacts. In general, the developed microwave-assisted solvent-free procedure for preparation of aromatic amides in good yields using a simple and efficient approach may find broad applicability in synthesis of various substrates containing amide functions. 

## Supplementary Materials

Supplementary materials can be accessed at: http://www.mdpi.com/1420-3049/17/2/1292/s1.
